# Physiological markers of anxiety are increased in children of abused mothers

**DOI:** 10.1111/j.1469-7610.2011.02410.x

**Published:** 2011-04-19

**Authors:** Tanja Jovanovic, Ami Smith, Asante Kamkwalala, James Poole, Tara Samples, Seth D Norrholm, Kerry J Ressler, Bekh Bradley

**Affiliations:** 1 Emory University School of Medicine, Dept of Psychiatry and Behavioral SciencesAtlanta, GA, USA; 2 Atlanta VA Medical Center, Mental Health ServiceDecatur, GA, USA; 3 Howard Hughes Medical InstituteBethesda, MD, USA; 4 Yerkes National Primate Research CenterAtlanta, GA, USA; 5 Fielding Graduate UniversitySanta Barbara, CA, USA

**Keywords:** Child abuse, maternal trauma, child anxiety, startle response, heart-rate variability

## Abstract

**Background:**

A growing number of studies indicate that low income, African American men and women living in urban environments are at high risk for trauma exposure, which may have intergenerational effects. The current study employed psychophysiological methods to describe biomarkers of anxiety in children of traumatized mothers.

**Methods:**

Study participants were recruited from a highly traumatized urban population, comprising mother–child pairs (*n* = 36) that included school-age children. Mothers were assessed for childhood abuse with the Childhood Trauma Questionnaire, as well as symptoms of depression and posttraumatic stress disorder (PTSD). The children were measured for dark-enhanced startle responses and heart-rate variability.

**Results:**

Dark-enhanced startle was found to be higher in children whose mothers had high levels of childhood physical abuse, as compared to children whose mothers had low levels of physical abuse. During the habituation phase of the startle experiment, children whose mothers had high levels of childhood emotional abuse had higher sympathetic system activation compared to children of mothers with low emotional abuse. These effects remained significant after accounting for maternal symptoms of PTSD and depression, as well as for the child’s trauma exposure.

**Conclusion:**

These results demonstrate that children of mothers who have history of childhood physical and emotional abuse have higher dark-enhanced startle as well as greater sympathetic nervous system activation than children of mothers who do not report a history of childhood physical and emotional abuse, and emphasize the utility of physiological measures as pervasive biomarkers of psychopathology that can easily be measured in children.

Childhood maltreatment has pervasive and detrimental neurobiological and psychological consequences; for recent review, see Heim, Shugart, Craighead, and Nemeroff (2010). Studies have suggested that abuse during childhood is associated with higher prevalence of adult mood and anxiety disorders (McCauley et al., 1997). The negative impact of early adverse events on the brain has been established for more than a decade: animal models as well as human clinical studies of early life stress have found long-term neurobiological effects (Carpenter et al., 2009; Heim et al., 2000; Plotsky & Meaney, 1993). Our laboratory has recently found that high levels of child abuse are associated with increased startle reactivity in adulthood (Jovanovic et al., 2009). Furthermore, different types of abuse (i.e., physical, sexual, emotional) appear to have differential effects on neurobiology; emotional and sexual, but not physical, abuse has been found to alter neuroendocrine function (Cicchetti, Rogosch, Gunnar, & Toth, 2010). On the other hand, we found that sexual and physical abuse, but not emotional abuse, were associated with psychophysiological changes (Jovanovic et al., 2009).

There is substantial evidence that maternal trauma exposure and subsequent psychopathology have transgenerational effects and are related to altered biological and psychological outcomes in their children. The effects of maternal trauma may be moderated by negative parenting behavior, parental psychopathology, shared trauma exposure, genetic or epigenetic risk (Grillon et al., 2005; Yehuda, Bell, Bierer, & Schmeidler, 2008). One study found that Holocaust survivors with post-traumatic stress disorder (PTSD) were more likely to engage in emotional abuse/neglect toward their children which, in turn, predicted alterations in their offspring’s PTSD symptoms (Yehuda, Halligan, & Bierer, 2001a; Yehuda, Halligan, & Grossman, 2001b). Another study which examined women exposed to September 11 WTC attacks during pregnancy, reported that trauma exposure altered hypothalamic–pituitary–adrenal (HPA) axis function in their infants, suggesting in utero transmission (Yehuda et al., 2005). A recent study examined the effects of maternal child abuse on their infants’ cortisol levels and found that children of mothers with a history of abuse had decreased baseline cortisol levels (Brand et al., 2010). Maternal behavior towards her child, such as increased impulsiveness, may mediate the relationship between the mother’s history of abuse and negative child outcomes (Möhler et al., 2009).

There is a small body of literature on physiological outcomes in children for whom maternal mental illness and/or trauma exposure place them at increased risk for psychopathology. One of the only longitudinal studies of such vulnerability traits in offspring of probands with anxiety disorders found that high-risk offspring had greater autonomic reactivity and increased startle responses compared to low-risk offspring (Merikangas, Avenevoli, Dierker, & Grillon, 1999; Monk et al., 2001). Grillon and colleagues (Grillon, Dierker, & Merikangas, 1997) found that greater startle magnitude delineated children of parents with anxiety disorders from children of parents with alcohol abuse or dependence. A recent multi-generational study of patients with major depression found that heightened startle magnitude was evident in the 3rd generation of the probands, showing a pervasive transgenerational effect (Grillon et al., 2005). Similarly, maternal psychopathology appears to increase sympathetic nervous system activity in their offspring (Dierckx et al., 2009).

Several peripheral psychophysiological measures have been used as indices of hyper-arousal and anxiety, including the acoustic startle response (ASR) and heart-rate variability (HRV). These measures offer non-invasive methods for assessing neurobiological activity, such as neural activity within the central limbic system, and the autonomic nervous system. The acoustic startle response is characterized by an integrative, reflex contraction of the skeletal musculature in response to a sudden intense stimulus (Landis & Hunt, 1939). It is mediated by a simple subcortical three-neuron circuit (Davis, 1992), but is modulated by limbic brain structures such as the amygdala in fearful or anxiogenic situations (Davis, 1992). For example, delivering startle probes in the dark reliably increases startle responses in humans (Grillon, Pellowski, Merikangas, & Davis, 1997). This effect of darkness is an analogue of light-enhanced startle in rodents, which is dependent on the limbic structure of the bed nucleus of the stria terminalis (Walker & Davis, 1996). The availability of such animal models and ability to capitalize on the understanding of underlying neurocircuitry is a great advantage of the startle measure relative to electrodermal measures such as skin conductance. Furthermore, dark-enhanced startle requires no training or delivery of aversive stimuli, and is thus more tolerable for pediatric populations. HRV measures autonomic nervous system control of cardiac activity and can be assayed across different frequency bands using power spectral analysis; the high-frequency band (HF-HRV) is a measure of parasympathetic, or vagal inputs to the heart. The ratio of the low-frequency to the high-frequency band (LF/HF HRV ratio) is a measure of sympathovagal balance. These measures provide more detailed insight into cardiovascular stress reactivity compared to heart-rate; they have frequently been measured in children and adolescents and have been associated with both acute anxiety (Arai et al., 2009; Hannesdóttir, Doxie, Bell, Ollendick, & Wolfe, 2010; Monk et al., 2001) as well as trait anxiety (Monk et al., 2001) and hyper-arousal (Greaves-Lord et al., 2007).

Based on the above-mentioned transgenerational neurobiological changes associated with maternal trauma, and our previous studies of altered startle reactivity in adults with a history of child maltreatment, we hypothesized that maternal child abuse history would be related to altered startle and autonomic nervous system responses in their children. This is the first study to examine these physiological markers in children of abused mothers; it is the first step to developing laboratory paradigms that measure phenotypes of risk in a highly traumatized population. This step is of great importance in assessing high-risk children for precursors of psychopathology that may lead to early interventions.

## Method

### Study sample and psychological assessment

Thirty-six children and their mothers participated in the study. The participants were recruited from the waiting rooms of the Primary Care, Obstetrics Gynecology or Child and Adolescent Psychiatry Outpatient Clinic (CAPOC) at the Grady Health System in Atlanta, GA. Inclusion criteria for the mothers were: 18–65 years of age, primary caretaker of a 6- to 13-year-old child, willing and able to sign informed consent; exclusion criteria were active psychosis, bipolar disorder, suicide ideation, and significant medical illness. Eligible child participants were between 6 and 13 years of age and willing to participate; exclusion criteria were autism spectrum disorders, bipolar or psychotic disorders, or cognitive disability. Prior to their participation, all mothers signed informed consent as well as parental permission for their children, and the children provided study assent approved by the Emory University Institutional Review Board and the Grady Research Oversight Committee.

The Structured Clinical Interview for DSM-IV (First, Spitzer, Williams, & Gibbon, 1995) was administered to all mothers. In addition to the diagnostic interview, all participants completed the Childhood Trauma Questionnaire (CTQ), the PTSD Symptom Scale (PSS), the Beck Depression Inventory (BDI), and the Traumatic Events Screening Inventory Parent Report (TESI). The CTQ is a self-report inventory assessing perceived childhood physical, sexual, and emotional abuse. Bernstein and Fink (1998) established scores for none, mild, moderate, and severe for each type of abuse. The data from the CTQ were used to classify participants into two categories for each type of abuse (physical, sexual, and emotional): (1) low abuse included those with CTQ scale scores in the ‘none to mild’ range, and (2) high abuse included those with CTQ scores in the ‘moderate to severe’ range. The PSS is a psychometrically valid 17-item self-report scale assessing PTSD symptomatology over the two weeks prior to rating (Falsetti, Resnick, Resick, & Kilpatrick, 1993). The PSS provides a continuous measure of PTSD symptom severity and has been validated with the Clinician Administered PTSD Scale (CAPS; (Foa, Riggs, Dancu, & Rothbaum, 1993; Foa & Tolin, 2000). The BDI consists of a 21-item questionnaire (Beck, Ward, Mendelsohn, Mock, & Erbaugh, 1961). This instrument provides a well-validated, commonly used, continuous score of depressive symptoms. The TESI is a 24-item parent-report version of a structured clinical interview that inquires about the child’s lifetime experience trauma exposure (Ribbe, 1996).

### Data acquisition and experimental design

The physiological data were acquired using Biopac MP150 for Windows (Biopac Systems, Inc., Aero Camino, CA). The acquired data were filtered, rectified, and smoothed using MindWare software (MindWare Technologies, Ltd., Gahanna, OH) and exported for statistical analyses. Startle data were collected by recording the eyeblink muscle contraction using the electromyography (EMG) module of the Biopac system. The startle response was recorded with two Ag/AgCl electrodes; one was placed on the orbicularis oculi muscle below the pupil and the other 1cm lateral to the first electrode. A common ground electrode was placed on the palm. Impedance levels were less than 6 kilo-ohms for each participant. The startle probe was a 108-dB(A)SPL, 40 ms burst of broadband noise delivered through headphones (Maico, TDH-39-P). The maximum amplitude of the eyeblink muscle contraction 20–200 ms after presentation of the startle probe was used as a measure of startle magnitude.

Heart-rate variability (HRV) data were acquired using the electrocardiogram (ECG) module of the Biopac system. ECG was recorded using two disposable Ag/AgCl electrodes pre-coated with electrolyte gel; one was placed on the right side of the upper torso, 1cm below the clavicle, and the second on the inside surface of the left wrist. Respiration was measured using a chest band transducer. HRV was quantified during one-minute intervals by spectral analysis of the time-sampled interbeat interval series, according to the methods recommended by the Society for Psychophysiological Research Committee on Heart Rate Variability (Berntson et al., 1997). The LF/HF HRV ratio was derived from high-frequency HRV sampled from 0.12 to 0.40 Hz and low-frequency HRV sampled from 0.04 to 0.12 Hz.

The experimental paradigm began with a 2-minute acclimation period during which no startle probes were delivered, followed by a startle habituation segment, and a dark-enhanced startle segment that occurred without interruption. The startle habituation segment consisted of two blocks with four startle probes in each block, for a total of eight probes. Immediately following habituation, participants underwent the dark-enhanced segment consisting of two blocks each with eight startle probes. In each block, four startle probes were delivered in the dark phase and four were delivered in the light phase. The light and dark phases alternated, with each phase lasting one minute. The order of light and dark was counterbalanced across subjects. The lights in the startle booth were controlled by a timer which was synchronized with the presentation of the startle probes. Across the entire experimental session, inter-trial intervals ranged from 9 to 22 seconds. The session duration was 8 minutes long (2 minutes acclimation, 2 minutes habituation, and 4 minutes dark-enhanced segment).

### Statistical analyses

The group variables in the analyses were derived from the abuse categories on the CTQ. For each type of abuse (physical, sexual, and emotional), subjects were divided into Low and High Maternal Abuse groups, according to the norms defined by Bernstein and Fink (1998). High Abuse included mothers who reported ‘moderate to severe’ levels of childhood abuse. If a mother reported high levels of abuse on more than one type of abuse, she was included in the High Abuse group for each category of abuse. The Low Abuse group included those with CTQ scale scores in the ‘none to mild’ range. We included the subjects with mild levels of abuse in the same category as those with no abuse due to the high prevalence of childhood trauma in this population.

Child demographic data such as age, and maternal clinical data such as PTSD and depression symptoms, were compared between the maternal abuse groups using analyses of variance (ANOVA). Baseline levels of startle magnitude and HRV during the habituation phase were compared between maternal abuse groups using 2-way mixed ANOVAs with Habituation Block (2 levels) as the within-subjects factor. The startle and HRV data during the dark-enhanced segment were analyzed in a 3-way mixed ANOVA, with Block (2 levels) and Phase (Light, Dark) as repeated measures factors and maternal Abuse (High, Low) as the between-groups factor. The dependent variables were startle magnitude and the LF/HF HRV ratio. Significant interactions were decomposed into respective univariate ANOVAs comparing diagnostic groups, with child sex and age used as covariates in the between-groups analyses of psychophysiological data in order to control for neurodevelopmental differences across age and between males and females (Gogtay et al., 2004). Finally, in order to examine the differential contributions of maternal abuse history and psychopathology to the dependent measures, we performed hierarchical regression analyses in which the child’s age and sex, maternal symptoms of PTSD (PSS score) and depression (BDI score), and maternal childhood abuse levels (CTQ scores for Physical, Emotional, and Sexual Abuse) were added at each step to predict startle magnitude and HRV indices. Separate regression analyses were conducted for each dependent variable (LF/HF HRV ratio and Dark-enhanced startle).

In the repeated measures ANOVAs, we used the Sphericity Assumed statistic to correct for violations of the sphericity assumption. All analyses were performed in SPSS 18.0 for Windows (SPSS, Inc.) with an alpha level of 0.05.

## Results

### Participant characteristics

Thirty-six (18 male, 18 female) children and their mothers participated in the study. The age of the children ranged from 6 to 13 years (M = 9.4, SD = 2.1). Of the 36 children, three stopped the session during the startle habituation phase, and another four children discontinued after the first dark-enhanced segment. The seven children that did not complete the startle sessions were younger than those that did complete (*F*(1,34) = 4.14, *p* = 0.05), but did not differ from the other 29 children on any other variables. Therefore, the final sample for the results of the habituation data and the first block of the dark-enhanced segment was 33, and for the second block of the dark-enhanced segment 29.

Three sets of analyses were performed – one for each type of abuse (Low vs. High Physical Abuse, Low vs. High Emotional Abuse, and Low vs. High Sexual Abuse). Table 1 shows descriptive and clinical data for each analysis. Across all abuse types, the children in the Low Abuse categories were younger than those in the High Abuse categories (Physical Abuse, *F*(1,32) = 5.78, *p* = 0.02; Emotional Abuse, F(1,32) = 7.96, *p* < 0.01; Sexual Abuse, *F*(1,32) = 6.41, *p* = 0.02). The distribution of boys and girls did not differ significantly between groups. As expected, the mothers in the High Abuse groups had significantly higher symptoms of PTSD and depression than mothers in the Low Abuse groups (Physical Abuse, PSS: *F*(1,32) = 5.79, *p* = 0.02, BDI: *F*(1,32) = 6.69, *p* = 0.02; Emotional Abuse, PSS: *F* (1,32) = 6.28, *p* = 0.02, BDI: *F* (1,32) = 13.06, *p* = 0.001; Sexual Abuse, PSS: *F* (1,32) = 15.89, *p* < 0.001, BDI: *F* (1,32) = 11.13, *p* = 0.002). In order to account for possible moderating effects of these variables, we performed stepwise regression analyses (see below).

**Table 1:** Demographic and clinical data for the sample, divided between childhood abuse groups

	Child demographics		Maternal psychopathology	
		
Maternal Abuse Group	Age, M ± SD	Sex, % female	PTSD, M ± SD	Depression, M ± SD
Physical Childhood Abuse				
Low (*n* = 22)	8.9 ± 2.0	54.5	11.5 ± 11.0	17.3 ± 10.0
High (*n* = 11)	10.7 ± 2.0*	45.5	22.3 ± 13.9*	28.5 ± 14.7*
Emotional Childhood Abuse				
Low (*n* = 24)	8.9 ± 2.1	54.2	11.9 ± 10.7	16.7 ± 9.7
High (*n* = 9)	11.1 ± 1.4*	44.4	23.7 ± 15.0*	32.2 ± 13.6**
Sexual Childhood Abuse				
Low (*n* = 17)	8.6 ± 2.2	52.9	7.7 ± 7.9	14.8 ± 8.5
High (*n* = 16)	10.4 ± 1.8*	50.0	22.7 ± 12.8**	27.7 ± 13.3**

The High and Low Maternal Abuse groups differ significantly in age of the child and the degree of maternal psychopathology. Childhood trauma was assessed using the Childhood Trauma Questionnaire (CTQ); PTSD symptoms were assessed using the PTSD Symptom Scale (PSS); Depression symptoms were assessed using the Beck Depression Inventory (BDI).

* *p* < 0.05;

** *p* < 0.01.

### Physiological data: habituation phase

A repeated measures ANOVA of startle magnitude across the two Habituation Blocks and between High and Low Physical Abuse groups showed no effect of Block, no main effect of Abuse group, and no interaction effect. The same was true for Emotional and Sexual Abuse groups. On the other hand, the analysis of LF/HF HRV ratio across the two Habituation Blocks revealed a significant interaction effect of maternal Physical Abuse and Block, *F* (1,31) = 6.26, *p* = 0.02, but no main effects of Block or Abuse. High and Low Emotional Abuse also interacted significantly with Habituation Block on LF/HF HRV ratio, *F* (1,31) = 7.09, *p* = 0.01. Maternal Sexual Abuse was not associated with LF/HF HRV ratio either as an interaction effect or as a main effect.

Follow-up univariate analyses of LF/HF HRV ratio within each Habituation Block demonstrated that children of mothers with High Physical Abuse tended to have greater ratios (M = 1.88, SE = 0.48) than children of mothers with Low Physical Abuse (M = 0.68, SE = 0.33) during the first Block, *F* (1,32) = 3.88, *p* = 0.06. Similarly, children of mothers with High levels of Emotional Abuse had significantly higher ratios (M = 2.31, SE = 0.53) than those of mothers with Low levels of Emotional Abuse (M = 0.62, SE = 0.31) during the first Block, *F* (1,32) = 7.10, *p* = 0.01. There were no group differences during the second Habituation Block. We repeated this analysis using a univariate ANCOVA with child demographics (age and sex), maternal psychopathology (PSS and BDI), as well as child trauma exposure on the TESI, as covariates, and the effect of maternal abuse remained significant, *F*(1, 26) = 5.29, *p* = 0.03. These results suggest that children of mothers with a history of high levels of abuse have greater sympathetic nervous system activation relative to parasympathetic activation.

In order to examine the differential contributions of maternal abuse and psychopathology on the above effects, we entered child demographics, maternal psychopathology, and maternal abuse history as predictors in a stepwise hierarchical regression model. In the first analysis, the dependent variable was the LF/HF HRV ratio during the first block of Habituation. Table 2 shows the results of the regression analysis, which showed that after controlling for other variables, maternal Emotional Abuse accounted for 20.6% of the variance in HRV, *F*_change_(1,25) = 9.48, *p* = 0.005, while neither Physical nor Sexual Abuse had significant effects. In order to examine the effect of the child’s own trauma exposure, we repeated the regression analyses and added maternal report of the child’s exposure to trauma (TESI) in the final step. This analysis revealed an even stronger relationship between maternal childhood Emotional Abuse and increased sympathetic activation after controlling for the child’s trauma *F*_change_(1,20) = 14.88, *p* = 0.001.

**Table 2:** Regression analysis of predictors of increased sympathovagal balance

Habituation LF/HF HRV ratio Model	*R*^2^	*R*^2^ change	*F* Change	*p*
1. Child’s demographics	0.02	0.02	0.34	0.71
2. Maternal psychopathology	0.20	0.18	2.95	0.07
3. Maternal childhood physical abuse	0.25	0.05	1.80	0.19
4. Maternal childhood emotional abuse	**0.46**	**0.21**	**9.48**	**0.005**
5. Maternal childhood sexual abuse	0.46	0.00	0.02	0.90

Hierarchical regression analyses examining the contributions of child demographics (age, sex), degree of maternal psychopathology (PTSD, MDD), and level of each type of maternal childhood abuse on LF/HF HRV ratio in children during the first block of the experiment. Abbreviations: PTSD: posttraumatic stress disorder; MDD: major depression disorder; LF/HF HRV: low frequency to high frequency heart-rate variability.

### Physiological data: dark vs. light phase

A 3-way repeated measures ANOVA of startle magnitude across the two Dark-enhanced Blocks (2 levels) as well as Phase (Dark vs. Light), between High and Low Physical Abuse groups, showed a trend level effect of Block, *F* (1,27) = 3.32, *p* = 0.08, and a significant main effect of Phase, *F* (1,27) = 14.19, *p* = 0.001, as well as a significant interaction effect of Phase and Physical Abuse, F(1,27) = 4.86, *p* = 0.04, see Table 3. For the other two types of abuse, there was also a significant effect of Phase, but no interaction of maternal Abuse group and Phase. On the other hand, the analysis of LF/HF HRV ratio across the two Dark-enhanced Blocks and Phase revealed a significant 3-way interaction effect of maternal Physical Abuse and Phase and Block, *F* (1,26) = 4.13, *p* = 0.05, but no main effects of Block, Phase, or Abuse. High and Low Emotional Abuse also showed a 3-way interaction on LF/HF HRV ratio, *F* (1,26) = 4.03, *p* = 0.05. Maternal Sexual Abuse was not associated with LF/HF HRV ratio either as an interaction effect or as a main effect. The 3-way analyses were followed up by 2-way ANOVAs within each Block, given that four children discontinued the session after the first block.

**Table 3:** Child startle results across experimental conditions (Dark vs. Light Phase) in the first block and groups (High vs. Low Maternal Abuse)

	Child startle magnitude (μV)				Effect		
		
	Light		Dark				
					
Maternal Abuse Group	*M*	SE	*M*	SE	Phase	Abuse	Phase × Abuse
Physical Childhood Abuse					*p* = 0.01	ns	*p* = 0.02
Low (*n* = 22)	40.7	9.0	45.4	8.7			
High (*n* = 11)	46.3	12.7	75.1	12.4			
Emotional Childhood Abuse					*p* = 0.01	ns	*p* = 0.04
Low (*n* = 24)	43.6	8.6	50.3	8.7			
High (*n* = 9)	39.8	14.0	68.7	14.2			
Sexual Childhood Abuse					*p* = 0.05	ns	ns
Low (*n* = 17)	44.4	10.2	51.3	10.5			
High (*n* = 16)	40.6	10.5	59.6	10.8			

Startle magnitude was greater in the dark phase; for physical and emotional abuse, there was a significant Phase × Abuse group interaction.

**Figure 1:**
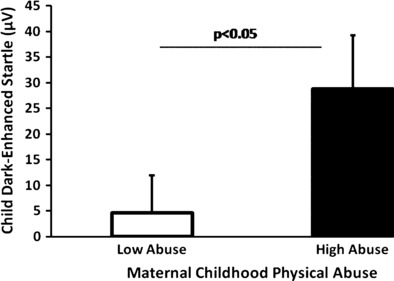
Mean dark-enhanced startle (difference score between Dark and Light Phases) during the first block in children of mothers with High and Low Physical Abuse

A 2-way analysis of Phase (Dark vs. Light) and maternal Physical Abuse during the first Block revealed a significant interaction effect, *F*(1,29) = 5.80, *p* = 0.02, with children of High Abuse mothers showing a greater increase in startle during the Dark Phase compared to children of mothers with Low levels of abuse. There were no group or interaction effects in the second block. The 2-way interaction was followed up by examining the relative increase during the Dark vs. Light phase in each group. In order to compare the change in startle magnitude between groups and control for individual differences in startle response, we calculated a difference score (startle during Dark Phase minus startle during Light Phase) for the first block. A univariate analysis of the difference score between Abuse groups found a significantly greater difference between Dark and Light in the High Abuse group compared to the Low Abuse group, *F* (1,32) = 6.00, *p* = 0.02 (see Figure 1). We repeated this analysis using a univariate ANCOVA with child demographics (age and sex), maternal psychopathology (PSS and BDI), as well as child trauma exposure on the TESI, as covariates, and the effect of maternal abuse remained significant, *F*(1, 26) = 6.61, *p* = 0.02. Two-way ANOVAs of Phase and Abuse on LF/HF HRV ratio separately within each Block of the Dark-enhanced segment found no significant interaction or main effects of either Physical or Emotional Abuse.

A regression analysis using the same predictors as above and the difference score between the Dark and Light phase as the dependent variable was performed. Table 4 shows the results of this analysis, with maternal Physical Abuse alone accounting for 16% of the variance in Dark-enhanced startle, *F*_change_(1,26) = 5.55, *p* = 0.03, while neither Emotional nor Sexual Abuse had significant effects. Again, the child’s trauma exposure strengthened the association between maternal trauma and the child’s Dark-enhanced startle, *F*_change_(1,20) = 8.81, *p* = 0.008. Since controlling for the child’s trauma did not reduce or eliminate the effect on child’s startle, these data suggest that maternal, rather than child, trauma is accounting for the observed effect.

**Table 4:** Regression analysis of predictors of increased dark-enhanced startle

Dark-enhanced startle MODEL	*R*^2^	*R*^2^ change	*F* change	*p*
1. Child’s demographics	0.03	0.03	0.52	0.60
2. Maternal psychopathology	0.09	0.06	0.85	0.44
3. Maternal childhood physical abuse	**0.25**	**0.16**	**5.55**	**0.03**
4. Maternal childhood emotional abuse	0.25	0.00	0.04	0.84
5. Maternal childhood sexual abuse	0.32	0.06	2.20	0.15

Hierarchical regression analyses of potential predictors of increased dark-enhanced startle, examining the contributions of child demographics (age, sex), degree of maternal psychopathology (PTSD, MDD), and level of each type of maternal childhood abuse on dark-enhanced startle in children during the first block of the experimental session. Abbreviations: PTSD: posttraumatic stress disorder; MDD: major depression disorder.

## Discussion

In this study of children growing up in low-income urban environments at high risk for trauma exposure and subsequent psychopathology, maternal childhood physical abuse was related to increased dark-enhanced startle in their offspring. In addition, childhood emotional abuse of the mother predicted increased sympathetic nervous system activation to the startle probe in her children. On the other hand, maternal sexual abuse was not associated with psychophysiological markers in the children. Importantly, the relationship between maternal abuse and child psychophysiology was not accounted for by either maternal psychopathology or the child’s own trauma exposure.

A growing body of literature suggests familial aggregation of anxiety disorders (e.g., (Cooper, Fearn, Willetts, Seabrook, & Parkinson, 2006; Last, Hersen, Kazdin, Orvaschel, & Perrin, 1991), as well a ‘vicious cycle’ of abuse from one generation to the next (Egeland, Jacobvitz, & Sroufe, 1988). Intergenerational transmission of psychopathology has been found in several studies examining the effects of maternal PTSD (Yehuda et al., 2001a, 2001b) and depression on the presence of anxiety symptoms in first and even second generation offspring (Warner, Wickramaratne, & Weissman, 2008). This transmission likely involves both behavioral and biological mechanisms; for example, mothers with a history of child abuse show alterations in their responses to their own children (Möhler et al., 2009) as well as child-relevant stimuli (Casanova, Domanic, McCanne, & Milner, 1994). Such disruptions in the interactions between abused mothers and their children could result in heightened child anxiety. On the other hand, biological mechanisms are implicated in studies showing that trauma exposure in mothers alters HPA axis function in their children (Brand et al., 2010; Yehuda & Bierer, 2008; Yehuda et al., 2005), which has been found to be a risk factor for developing PTSD (Yehuda, 2002). Aside from this small number of neuroendocrine findings, no other research studies that we are aware of have found effects of maternal trauma on physiological markers of anxiety in their children.

The physiological markers that were affected by maternal trauma in this study, namely, dark-enhanced startle and LF/HF HRV ratio, have been associated with arousal and anxiety. Dark-enhanced startle is the human analogue of light-enhanced startle in rodents, which is dependent on nuclei in the limbic system (Walker & Davis, 1996). This startle index is elevated in adults with PTSD and trauma survivors without PTSD (Grillon, Morgan, Davis, & Southwick, 1998) as well as children at risk for anxiety disorders (Grillon, Dierker et al., 1997). LF/HF HRV ratio is related to the balance between the sympathetic and parasympathetic components of the autonomic nervous system – in addition to being an acute measure of arousal, this is also an index of cardiovascular health (Monk et al., 2001). It is possible that maternal trauma increases risk of negative mental and physical health outcomes in their children. A recent meta-analysis of HRV studies suggests that sympathetic drive is higher than parasympathetic tone in anxiety disorders (Cohen & Benjamin, 2006), a finding that is consistent with heightened LH/HF HRV ratio as a marker of anxiety in our study of children of abused mothers. We found that this measure was specifically associated with high levels of emotional abuse experienced by the mother in her childhood, while dark-enhanced startle was related to physical maternal abuse. There is an emerging body of research on the ways in which specific types of childhood abuse demonstrate differential effects on neurobiology (Cicchetti et al., 2010). For example, Carpenter and colleagues (Carpenter et al., 2009) found that emotional abuse and age at time of abuse predicted reactivity to cortisol in adulthood. In our previous study of adults with a history of childhood abuse, we found that sexual and physical abuse, but not emotional abuse, increased startle responses (Jovanovic et al., 2009).

Dark-enhanced startle was significantly elevated in offspring of abused mothers during the first block of the session, but not the second block. The discrepancy between Block 1 and Block 2 could be due to a tendency for the startle response to habituate from one block to the next; although the average startle magnitude in the dark was still greater than the light in the second block, overall all startle responses were reduced. It is possible that the initial impact of darkness has the greatest effect on startle. Notably, the four children who discontinued the session did not account for the effect of darkness in the first block. The effect remained even after the four children were removed from the analysis of the first block. The advantage of the main effects being present in the initial phase of the experiment is that the session could be potentially shortened, thereby making it even more tolerable to children.

It is important to note that maternal PTSD and depression symptoms, while increased in the mothers with high compared to low levels of childhood abuse, did not account for the increased physiological activation in their children. After controlling for maternal psychopathology, it was maternal trauma that predicted the children’s responses. Furthermore, the effects were also not accounted for by the child’s own trauma exposure. Given that mothers with childhood trauma may raise their children in environments that increase risk of trauma exposure, this would be a likely candidate for the effects of intergenerational transmission of psychopathology. However, the maternally reported trauma exposure of the child did not contribute to the child’s physiological responses. Clearly, a limitation of the study is reliance of maternal report for the measure of child trauma levels. Specifically, it is possible that mothers withheld information about overly harsh or abusive parenting. Therefore, one possible explanation for our data is that maternal history of abuse may have led to increased levels of harsh verbal and/or physical punishment towards the child resulting in increased stress reactivity (as indicated by increased dark-enhanced startle and increased sympathetic nervous system activation to the startle probe). Our future research will include interviews with the children in the absence of the mothers, to get a more direct measure of child trauma exposure.

A limitation of the study is the use of a retrospective self-report measure of child abuse. While this instrument has been validated in previous studies (Bernstein et al., 2003; Bradley et al., 2008), there may be inherent reporting bias in adult assessments of childhood trauma history. Given that we do not have longitudinal, objective measures of child abuse we are relying on the subjects’ perception of their abuse history. However, given the high rates of trauma exposure, substance abuse, and history of incarceration in this low-income population, it is likely that the reported abuse is accurate. The CTQ has shown very good convergence with other measures of child abuse, indicating that it is a valid instrument (Bernstein et al., 1994). Furthermore, this subjective perception may in fact be related to individual sensitivity, which in itself may be a risk factor for psychopathology. In order to tease these contributing factors apart, future studies may incorporate a more direct measure of child abuse and utilize prospective approaches.

Additional limitations of the study are the small sample size, and the lack of inclusion of clinical anxiety measures for the children. Follow-up studies will collect these measures to see if they correlate with the physiological indices. Of interest will also be to collect measures of sleep quality in the children to see whether darkness has relevance in their daily functioning. Here we present our preliminary and highly novel results of the association between maternal trauma and child physiological markers that have been previously associated with anxiety (Dierckx et al., 2009; Grillon, Dierker, and Merikangas, 1997a; Monk et al., 2001; Waters et al., 2008). The low-income African American population under investigation is at high risk for both trauma exposure and anxiety disorders. Anxiety disorders begin to develop in children as young as 5 years old (Paus, Keshavan, & Giedd, 2008) – investigating early biomarkers of these disorders is a high research priority in the field of psychiatry.

## Key points

- Prior studies have found that heightened dark-enhanced startle responses and dysregulated sympathovagal balance are associated with anxiety in adults and children.
- The current study found that maternal childhood physical abuse was associated with heightened dark-enhanced startle in their children during the initial phase of the experiment.
- The current study found maternal childhood emotional abuse was associated with heightened sympathetic nervous system activity in their children.
- The effects of maternal childhood trauma were not accounted for by maternal PTSD or depression, or the child’s own trauma exposure.
- This is the first study to find transgenerational effects of maternal trauma on psychophysiological markers of anxiety in children.
